# Percutaneus Mitral Balloon Valvulotomy Using Left Femoral Vein; an Unusual Case

**Published:** 2013-06-01

**Authors:** Mahmoud Ebrahimi, Ali Eshraghi

**Affiliations:** 1 Department of Cardiology, Imam Reza Hospital, Mashhad, IR Iran

**Keywords:** Balloon Valvuloplasty, Femoral Vein, Mitral Valve Stenosis

## Abstract

Balloon valvulotomy is the procedure of choice for relief of mitral stenosis. Inoue technique is the most widely accepted technique which uses the right femoral vein to access the interatial septum. Due to technical issues, left femoral vein approach is less recommended.

We report an unusual case in which the right femoral vein was not accessible and mitral balloon valvulotomy was done via left femoral approach.

Left femoral vein may be used in special cases where the right femoral vein is not accessible.

## 1. Introduction

Mitral Stenosis (MS) is a frequent cause of morbidity and mortality, especially in developing countries.

The predominant cause of MS is rheumatic fever (1).

Clinical presentations of MS include dyspnea, hemoptysis, chest pain, palpitation, and embolic events (2).

Moreover, severe symptomatic MS is associated with poor long-term outcomes and mechanical intervention is required (3).

Percutaneous Balloon Mitral Valvulotomy (BMV) is the procedure of choice for treatment of MS. Besides, surgical intervention is reserved for the patients who are not candidate for percutaneous procedure (4).

In general, BMV is recommended for symptomatic patients with moderate to severe MS and favorable valve morphology or when valve morphology is not ideal, but the patients are at a high risk for surgery. However, BMV is not recommended for the cases with more than mild mitral regurgitation or evidence of left atrial thrombus (3).

Currently, BMV is most often performed using transsepteal access to the left atrium.

Inoue balloon is the most frequently used balloon inflatable catheter for MS (5). Wilkins scoring system is used to determine the eligibility of the valves for BMV. A score below 8 indicates favorable response to the percutaneous procedure (5).

Inoue balloon technique has limited complications, but durable benefits (5).

In BMV, transseptal puncture is performed from the right atrium via the femoral vein. Technically, the right femoral vein is more appropriate and most widely used (6). Nevertheless, the left femoral vein is not preferred because of technical issues, such as difficult passage of Brockenbrough needle and dilator through the left iliac vein and inferior vena cava acute angle and difficulty of correct puncture site location (7). Nonetheless, there are few unusual cases in which the right femoral vein cannot be accessed and, consequently, another route has to be selected.

## 2. Case Report

Our patient was a 62-year-old male, 175 cm tall who complained about dyspnea on exertion (NYHA class III). The patient was on beta blocker, warfarin, and diuretic and his rhythm was atrial fibrillation. Transthoracic and transesophageal echocardiography was performed and severe mitral stenosis was established. Mitral valve area was 0.8 cm^2^ and no thrombus was found in the left atrium and its appendage. In addition, Wilkins score was 6 and systolic pulmonary artery pressure was 38 mmHg.

The patient was candidatedfor BMV.

Before attempting for valvulotomy, coronary artery status was determined. Selective coronary angiography demonstrated no significant obstruction in the coronary arteries. First, the right femoral vein was approached, but it could not be accessed; therefore, the left femoral vein was punctured. Then, the retrograde injection of the contrast media in the proximal of the right external illiac vein showed that the right femoral vein was occluded. Thus, we decided to continue the procedure from the left side.

Transseptal puncture of the interatrial septum was performed via the left femoral vein using Brocken Brugh needle. After dilatation of this puncture site, Inoue balloon 28 was used and graded inflation was done with 24, 26, and 28 cc. The mean transmitral valve gradient was declined from 16 mmHg to 3 mmHg and the left ventricudar injection revealed no mitral regurgitation. After all, the procedure was terminated without any complications.

The patient was discharged two days after the procedure with beta blocker and anticoagulant (warfarin) due to atrial fibrillation rhythm.

## 3. Discussin

In developing countries such as Iran, rheumatic heart disease is prevalent and its consequences, such as MS, are major health issues.

Due to the prevalence of rheumatic heart disease, unusual cases of valvular involvement and challenging therapeutic options are not infrequent. Therefore, practicing physicians in these countries must be trained in managing the extraordinary cases.

Inoue procedure in BMV is a familiar and popular technique for interventional cardiologists in Iran. Traditionally, the right femoral vein was used for this procedure because of technical issues (6) . This route is so popular that inability to access the right femoral vein may be considered as one reason to hesitate the BMV procedure.

In reviewing the published medical literature, only a few reports were found regarding the use of left instead of right femoral vein.

In 1995, Patel et al. reported one case of BMV from the left side (7).

In 2008, Tchavdar N Shalganov et al. reported using the left femoral vein for transseptal access to the left atrium for electrophysiological study purposes (8).

In another case report, Namboodiri et al. reported left femoral approach for BMV in a case of mirror image dextrocardia (9).

Vyas et al. also used this unusual approach in 2011 (10).

These studies illuminate a new approach for rare cases in whom, the traditional Inoue technique from the right femoral vein is not applicable.

Another important feature of our patient is his age.

Regarding 15-20 years necessary time from onset of rheumatic heart involvement to developing significant MS (3), we may think that the patient has had rheumatic heart involvement in the 4th decade of his life which is quite unusual.

## 4. Conclusion

In cases which need transseptal access to the left atrium and the right femoral vein is not accessible, the left sided approach is feasible and approachable.
